# Correlation of Anti-Mullerian Hormone Level and Antral Follicle Count with Oocyte Number in A Fixed-Dose Controlled Ovarian Hyperstimulation of Patients of *In Vitro* Fertilization Program

**DOI:** 10.22074/IJFS.2021.6238

**Published:** 2021-01-27

**Authors:** Wiryawan Permadi, Mohammad Wahyu Ferdian, Dian Tjahyadi, Wulan Ardhana Iswari, Tono Djuwantono

**Affiliations:** Department of Obstetrics and Gynecology, Faculty of Medicine, Universitas Padjadjaran - Dr Hasan Sadikin Hospital (RSHS), Bandung, Indonesia

**Keywords:** Anti-Mullerian Hormone, *In Vitro* Fertilization, Ovarian Response

## Abstract

**Background:**

This study was conducted to determine the correlation of anti-Mullerian hormone (AMH) level and antral follicle count (AFC) with oocyte count in women who had received controlled ovarian hyperstimulation in an *in vitro* fertilization (IVF) program.

**Materials and Methods:**

We retrospectively gathered the data of 42 patients who underwent IVF during 2005-2017 at Aster Clinic in Dr. Hasan Sadikin Hospital and Bandung Fertility Center Limijati Hospital, Indonesia. Details of the subjects such as identity, characteristics, history of illness, history of previous therapy, levels of ovarian reserve markers examined (AFC and AMH), follicle-stimulating hormone (FSH) dose given, and number of oocytes produced were recorded.

**Results:**

A significant positive correlation between AMH (P≤0.001, r=0.530), AFC (P≤0.001, r=0.687), and AMH- AFC combination (P≤0.001, r=0.652), and the number of oocytes was found at the FSH dose of 225 IU.

**Conclusion:**

AFC and AMH are able to reliably predict ovarian response to FSH.

## Introduction

Primary infertility affects 8-12% ofreproductive-age couples globally, and the proportion is estimated to vary from 4.5 to 30% across countries, with the highest percentages found in developing countries (1). In Indonesia, it was estimated that 12.3% of reproductive-age couples suffered from infertility, whereas another survey estimated a prevalence rate of 10-15% (2). As such, the demand for assisted reproductive technology, such as in *vitro* fertilization (IVF) has risen in recent years.

Adequate follicle growth, achievable through follicle stimulating hormone (FSH) administration in controlled ovarian hyperstimulation protocols, is crucial for the success of an IVF cycle (3, 4). However, the varieties inindividual characteristics and response to FSH stimulation among infertile patients havemade it difficult to generate a dose cut-off applicable for both low-responders and highresponders while avoiding the risk of ovarian hyperstimulation syndrome. FSH in an IVF cycle is therefore generally administered based on a fixed dose, and the standard dose used in our clinic was 225 IU. The two best known ovarian reserve markers to predict ovarian response to FSH are mean antral follicle count (AFC) and anti-Mullerian hormone (AMH), although there is a lack of data to conclude which of the two markers served better to predict ovarian reserve (5, 6). AFC is the number of follicles measuring 2-10 mm in size from both ovaries. AMH is detected in the primordial follicle and achieves peak level in the small antral follicle. The AMH level indicates the number of growing follicles, and this level can be used to determine the prognosis of fertility. The number of oocytes obtained will be probably low if the predicted AMH level is low, whereas extreme ovarian response complications can be expected when the predicted AMH level is excessive. Currently available ovarian reserve markers, including the AMH and AFC, invariably still show varied results, especially in clinical practice where AMH and AFC level could be at odds with each other (5-7).

It has been previously suggested that ethnicity may influence ovarian reserve markers (8-10). Furthermore, ethnicity may influence the manner with which ovarian reserve markers interact with factors such as age and weight. Age is negatively correlated with AMH and AFC in Caucasian, African-American, Hispanic, and Asian women, however BMI was only negatively correlated with serum AMH level in Caucasian women (8). A cross-sectional study comparing Indian and Spanish women showed that AFC is declined in younger Indian women compared to Spanish women (11). To date, there are few studies that have examined ovarian reserve markers in Indonesian women, much less inthose who received controlled ovarian hyperstimulation in an IVF program. Therefore, it is necessary to study the association of AMH and AFC to obtain an optimal ovarian response in this population. This study was conducted to determine the correlation of AMH and AFC with the number of oocytes in women who hadreceived controlled ovarian hyperstimulation in an IVF program with an FSH dose of 225 IU, which is the most frequently used dose in Indonesian health facilities.

## Materials and Methods

In this retrospectively study, the data were obtained from the medical records of patients who underwent the IVF program with the FSH dose of 225 IU at Aster Clinic, Hasan Sadikin Hospital, and Bandung Fertility Center Limijati Hospital, Indonesia. The sample size in this study was calculated by a sampling formula for unpaired analytic categorical study, set at α=0.5 and 1-β=90%. Proportion of the population (P_1_ and P_2_) were assumed to be 50 and 10%, respectively. The formula yielded a minimum of 26 samples. The inclusion criteria were patients who underwent the IVF program, aged ≤40 years, were given a constant exogenous FSH dose throughout the cycle, and whose medical record included complete patient characteristics, physical examination, AMH and AFC levels throughout the cycle. AMH and AFC measurements were done on the second or third day of the menstrual cycle and this was done consistently. The exclusion criteria were the presence of a history of ovarian surgery, polycystic ovary syndrome, endometriosis, ovarian cyst, orendocrine disease. We also recorded the patients’ identity, characteristics, previous medical history, previous medical therapy, levels of ovarian reserve markers (AFC and AMH), and the number of oocytes produced (the oocyte numbers in this study represent numbers for all oocytes aspirated). In this study, we selected patients as a whole, which means that all patients underwent the same treatment regimen using a short protocol with recombinant FSH and human chorionic gonadotropin, and all sperm used had normal parameters. 

### Ethics approval

This study protocol was approved by Faculty of Medicine, Universitas Padjadjaran, Ethics Committee Review Board (LB.04.01/ACS/TC/066/III/2018) and all study participants gave informed consent, patients consent to participate was written. All authors hereby declare that all patients have been examined in accordance with the ethical standards laid down in the 1964 Declaration of Helsinki.

### Statistical analysis

Numerical data arepresented as mean, SD, median and range. Data normality was assessed using the ShapiroWilk or Kolmogorov-Smirnov test. The subjects’ characteristics were compared using an unpaired t test or a Mann-Whitney test, as appropriate. Correlation between the variables was assessed using Pearson or Spearman correlation test. A P≤0.05 was considered statistically significant. SPSS v24.0 (IBM Corporation, USA) was used to perform statistical analysis.

## Results

Of 356 patients enrolled during 2005-2017, 42 patients met the study criteria. Mean age of the patients investigated in this study was 34.8 ± 2.8 years (range: 29-39 years, median: 35 years) and the body mass index (BMI) was 24.4 ± 4.1 kg/m^2^ (range: 14.3-33.3 kg/m^2^, median: 23.6 kg/m^2^). Baseline demographics of the study subjects are shown in Table 1. Normality test results showed that AFC and AMH-AFC levels were normally distributed, which were then analysed using Pearson correlation test, whereas AMH levels and oocyte amount were not normally distributed and analysed by Spearman correlation test.

**Table 1:** Baseline demographics and study subject characteristics


Variable	Statistical measure		
	Mean ± SD	Median	Range

Age (Y)	34.8 ± 2.8	35	29-39
BMI (kg/m^2^)	24.4 ± 4.1	23.6	14.3-33.3
AMH (ng/ml)	3.34 ± 2.28	2.66	0.50-9.90
AFC	9.81 ± 3.99	10	0.50-9.90
Oocyte retrieved	7.4 ± 3.4	7	2-22
AMH-AFC	13.1 ± 5.6	12.3	2.5-27.9


BMI; Body mass index, AMH; Anti-mullerian hormone, and AFC; Antral follicle count

The largest number of oocytes (4-15) was produced at a range of AMH of 1.2-4 ng/ml and AFC 4-15 (Table 2). There were 37 patients who produced 4-15 oocytes and they were classified as normo-responders. One patient was a hyper-responder due to the production of >15 oocytes; this excessive response was predicted as she had an AFC of >15. Furthermore, four patients produced <4 oocytes and they were classified as hypo-responders.

**Table 2:** Serum AMH and AFC according to ovarian stimulation response groups


Variable		Oocyte number		
		0-3	4-15	>15
		n=4	n=37	n=1

AMH				
	<1.2 ng/mL	1	4	0
	1.2-4 ng/mL	3	20	1
	>4 ng/mL	0	13	0
AFC				
	<4	1	1	0
	4-15	3	33	0
	>15	0	3	1


AMH; Anti-mullerian hormone and AFC; Antral follicle count.

AMH levels were analysed using Spearman correlation test, AFC and AMH-AFC were analysed by Pearson correlation test. A significant positive correlation was found between AMH (r=0.530, P≤0.001), AFC (r=0.687, P≤0.001), and AMH-AFC combination (r=0.652, P≤0.001) and the number of oocytes. To reduce bias from possible confounding by age, Spearman’s correlation was used to determine the correlation between age and number of oocytes. There was an insignificant negative correlation between age and number of oocytes produced (P=0.129 and r=-0.179). Pearson’s correlation test was used to determine the correlation between BMI and AMH, and between BMI and the number of oocytes retrieved; an insignificant positive correlation was found between the two variables (P=0.216, r=0.123, and P=0.452, and r=0.19, respectively).

## Discussion

Our study showed that AMH, AFC, and AMH-AFC are significantly correlated with the number of oocytes produced. This is in agreement with previous studies that investigated the relationship between AMH levels and the number of oocytes. Asada et a.l (12) observed a positive correlation between AMH level and oocyte count among Japanese women. AMH can effectively predict ovarian responses and allow clinicians to avoid iatrogenic complications and choose optimal stimulation strategies (13). However, AMH levels showed variations when examined by different examination kits and among different populations. Although we found a relatively strong and significant correlation between AMH levels and the number of oocytes with the FSH dose of 225 IU in this study, these potentially confounding factors should be considered.

Fertility begins to decrease at the age of 30 years and further decreases significantly after the age of 35 years (14, 15), which made our subjects’ age (34.8 ± 2.8) a significant potential confounder inour study. In the correlational analysis, however, we did not observe a significant interaction between age and the number of oocytes produced. 

How BMI influences ovarian reserve markers and the number of oocytes retrieved, is still unclear. In ametaanalysis, Moslehi et al. (16) concluded that AMH is significantly lower in obese women. On the other hand, a study of 402 women in Turkey, categorized based on ovarian reserve patterns (poor, <7 baseline AFC; adequate, ≥7 baseline AFC, and high ovarian reserve) and BMI group, revealed that serum AMH and FSH levels were similar across all categories (17). Another study of women receiving controlled ovarian stimulation for assisted reproductive technology reported that BMI did not negatively affect the number of oocytes retrieved (18). In our study, we did not find a significant correlation between BMI and AMH or BMI and oocyte count.

We observed a relatively strong positive correlation between AMH-AFC combination and the number of oocytes. In a sequential order, it can be observed that AFC correlates best with the number of oocytes, followed by AMH–AFC combination and AMH. The results of this study are in contrast to the result of a study conducted by Nelson et al. (19), which compared the predictive value of live births that indirectly represents the association between the number of oocytes and AMH, AFC, and AMH-AFC combination only with age, in the UK. The authors reported that AMH showed the best predictive value, followed by the combination of AMH-AFC and AFC only. In the present study, the strongest relationship was observed between the number of oocytes and AFC, and the results were not much different from those of the combination of AMH-AFC. This suggests that, in women without discordant ovarian marker, AFC may be a better choice compared to AMH in predicting ovarian reserve. This agrees with the results of Jayaprakasan et al. (20), who found that AFC predicts ovarian response better than AMH or a combination of AFC and AMH. This is further reinforced by the results of Liao et al. (21) whose study on 8269 women undergoing IVF/intracytoplasmic sperm injection (ICSI) treatment showed a strong association between AFC, number of oocytes retrieved, and clinical pregnancy rate. These data imply that in the absence of AMH examination, AFC may suffice, as it is wellcorrelated with the number of oocytes in clinical practice. AFC is easier, and relatively inexpensive, and offers almost immediate results. 

In the present study, we found a relatively strong positive correlation between AFC and the number of oocytes at the FSH dose of 225 IU. The AFC measurement performed using ultrasound was effective, easy to use, safe, and non-invasive. Therefore, estimating the number of antral follicles can be used as a predictive test of ovarian function, ovarian reserve, and ovarian response.

## Conclusion

Significant positive correlations of AMH levels, AFC, and AMH-AFC with the number of oocytes were found in this study. These correlations werestrong enough at the FSH dose of 225 IU. AFC is a better ovarian reserve marker compared to AMH and the combination of AMHAFC in predicting the number of oocytes. Existing data on variations in infertility causes and longevity suggest that an analysis free of infertility including confounding variables and duration, would be preferable. Our study limitations could be overcome by multivariable analysis, but a larger sample size is needed.
